# Editorial Change

**Published:** 1858-03

**Authors:** 


EDITORIAL CHANGE.—We congratulate the subscribers and friends—including ourselves among the latter—of the *Charleston Medical and Surgical Journal* upon the accession of Dr. J. Dickson Bruns to the editorial charge of that valuable periodical. Dr. Bruns is already favorably known to the reading portion of the profession, more especially as the author of an excellently written brochure entitled, "Life; Its Relations, Animal and Mental," published during the past year, and noticed in the January No. of this Journal.

